# Beyond the Impasse – Reflections on Dissociative Identity Disorder from a Freudian–Lacanian Perspective

**DOI:** 10.3389/fpsyg.2017.00789

**Published:** 2017-05-16

**Authors:** Reitske Meganck

**Affiliations:** Department of Psychoanalysis and Clinical Consulting, Ghent UniversityGhent, Belgium

**Keywords:** dissociative identity disorder, etiology, Freudian–Lacanian psychoanalysis, hysteria, treatment

## Abstract

Dissociative identity disorder (DID) is a widely contested diagnosis. The dominant posttraumatic model (PTM) considers early life trauma to be the direct cause of the creation of alter identities and assumes that working directly with alter identities should be at the core of the therapeutic work. The socio-cognitive model, on the other hand, questions the validity of the DID diagnosis and proposes an iatrogenic origin of the disorder claiming that reigning therapeutic and socio-cultural discourses create and reify the problem. The author argues that looking at the underlying psychical dynamics can provide a way out of the debate on the veracity of the diagnosis. A structural conception of hysteria is presented to understand clinical and empirical observations on the prevalence, appearance and treatment of DID. On a more fundamental level, the concept of identification and the fundamental division of human psychic functioning are proposed as crucial for understanding the development and treatment of DID.

## Introduction

James was 24 years old when he consulted a therapist, as his girlfriend discovered his adulterous behavior and a number of substantial purchases of which he wasn’t aware. He was confronted with sexually explicit and vulgar computer chats on his personal computer with another woman that indicated they had been meeting in real life as well. He was disgusted by the tone of the chats and the actions they described, and could not recognize himself in his online persona. However, he had to face the fact that it could be no one else but him. Also, credit card bills and electronic devices found in the basement evidenced excessive purchasing activity, yet he had no memory of having bought these items. James began to experience intense fear of not being able to trust himself, even when he was asleep. Apparently he had been sneaking out at night to meet another woman, waking up the next morning feeling tired, but without any recollection of what had happened.

This short clinical vignette presents a patient whose complaints reflect serious dissociative symptoms. The case presented was selected from our research program aiming at the systematic study of the process and outcome of psychoanalytic psychotherapy (Ghent University, Belgium). In the current paper, the author’s main focus is on the literature on dissociative identity disorder (DID), yet aspects of the case will be briefly returned to as an illustration of the addressed theoretical debates. Given the scope of this paper, this will necessarily comprise a limited account; for a more elaborate and systematic discussion of the case see Van Nieuwenhove et al. (unpublished).

The phenomenon of multiple personalities, currently diagnosed as DID, inspired popular imagination long before its inclusion in the Diagnostic and Statistical Manual of Mental disorders [DSM-II: hysterical neurosis, dissociative type (American Psychiatric Association, 1968); DSM-III: multiple personality disorder (American Psychiatric Association, 1980)]. The roots of this disorder as a psychiatric condition are to be found in France in the latter half of the 19th century. Around that time, starting from rising ideas on hypnosis, hysteria, and trauma, scientists studying patients presenting with seemingly different personalities, reinterpreted religious phenomena like possession and ecstasy in terms of unrecognized psychiatric problems (Baeten, 1998).

Currently, in the fifth edition of the DSM (DSM-V; American Psychiatric Association, 2013), DID is defined as a “disruption of identity characterized by two or more distinct personality states… (accompanied by) related alterations in affect, behavior, consciousness, memory, perception, cognition, and/or sensory-motor functioning… with recurrent gaps in the recall of everyday events, personal information and/or traumatic events” (p. 261). The main difference with the DSM-IV-TR (American Psychiatric Association, 2000) is the omission of the necessity for the diagnostician to observe the presence of different personality states him or herself (Spiegel et al., 2011). Across diagnostic systems, the most remarkable feature of DID is the existence of seemingly independent personalities with amnesia of the primary identity for the existence and behavior of (some) other identities (Putnam et al., 1986; Boysen and VanBergen, 2013). Given James’s apparent amnesia, and the observation that his behavior during dissociative states was radically uncharacteristic his usual functioning, he meets the DSM criteria for DID. At least two personality states were noted. While he was a polite, well-educated, and considerate person in his primary identity state, he was more aggressive, vulgar and sexually promiscuous in his dissociated personality state. Moreover, there seemed no reason to doubt his amnesia of this secondary state; his reactions of disgust (he vomited when confronted with the chats on his computer), intense fear and strong engagement in the therapeutic process, as well as the observations of his relatives, indicate that there are real memory gaps concerning this other personality state. Such distinct personality states are mostly called ‘alters’ in present-day literature. The term ‘alter’ refers to a so-called other personality state that takes over, and shows not only different behavior and personality characteristics, but sometimes also different clothing styles, another language, behaving of a different age… (Putnam et al., 1986; Kluft, 1988).

The clinical phenomenon of DID has always seized public fascination and to-date remains a highly controversial diagnosis. The controversy circles around two related issues, namely etiology and treatment. The issue of etiology concerns the question of the validity of the diagnosis, i.e., whether or not DID is real. Two opposing views are defended in the literature, the posttraumatic model (PTM; Putnam, 1989; Gleaves, 1996; Ross, 1997) and the socio-cognitive model (SCM; Spanos, 1994; Sarbin, 1997; Lilienfeld et al., 1999). The latter is also called the fantasy model because of its emphasis on fantasy proneness and suggestibility. As argued below, debate on the reality of the diagnosis is largely off topic as it seems that in the battle to prove or disprove the validity of the DID diagnosis, as either a disease or as a folly, both parties lose sight of the subject presenting these symptoms.

## Posttraumatic and Socio-Cognitive Models of DID

In the 1990s, fierce debate emerged on the veracity of the DID diagnosis (Pope et al., 2006). During this period, the water between traumatic models, on the one hand, and SCMs, on the other, was very deep: both were mainly trying to prove the extent to which the other party was wrong. Scanning the literature shows that often authors make caricatures of the views and arguments of the other party, thus polarizing the discussion into an infertile battle. Recently, a more nuanced appreciation of the different standpoints has been reached (e.g., Dalenberg et al., 2012; Boysen and VanBergen, 2013; Lynn et al., 2014) and both models seem to depict a more complex picture of the etiology of DID. However, as these publications indicate, the debate is far from settled. Below I outline the main points of these dominant models.

The first etiological approach was put forward by Janet (1889/1973), who considers DID as a distinct mental disorder, and dissociation as a defensive reaction to severe childhood trauma. Initially, this was considered to be sexual trauma, with occasional excursions to satanistic ritual abuse (Mulhern, 1994), but more recently disruptive experiences within (early) attachment relationships have come into focus (Schimmenti and Caretti, 2016). From the perspective of this PTM (e.g., Kluft, 1988; Putnam, 1989; Gleaves, 1996; Ross, 1997), dissociated states are presumed to be already present in children who use it as a way to avoid the trauma of abusive experiences. Here a direct link is made between the experience of trauma and dissociation, the latter being considered “a creative survival strategy that helped the individual to cope with overwhelming trauma” (Gleaves, 1996, p. 42). This is thought to potentially evolve into alter identities that are maintained in adulthood and express themselves when the individual has to cope with stressful situations and emotions disavowed by the primary identity. Accordingly, in the context of treatment there is an explicit focus on both the trauma and on the symptom of alters (International Society for the Study of Trauma and Dissociation [ISSTD], 2011). Several techniques are outlined to identify and work with alters, making them a core element in the therapeutic process.

The main argument put forward by PTM proponents is the strong association found between reported childhood trauma and DID symptoms and diagnoses. Indeed, in the case of James, we see a traumatic childhood: a dominant father who was both verbally and physically aggressive toward the children, and a mother who did not intervene at any point, even when her children were in danger. Critiques have been formulated concerning this association, which mainly address the cross-sectional nature of most existing studies, the scarcity of research using objective measures of trauma and the issue of false memories that previously gained attention in the United States (where the DID diagnosis is most common) (Spanos, 1994; Lilienfeld et al., 1999). Moreover, there is little evidence of dissociative identity conditions in children, a precondition within the PTM argument, which states that alters arise as a direct consequence of childhood trauma (Boysen, 2011)^1^.

The SCM proposes an iatrogenic origin (i.e., induced by treatment) to DID. In this respect, multiple identities are considered to be cultural role enactments or social constructions without a causal link with trauma (Spanos, 1994; Sarbin, 1995). The role of leading treatment models and therapist’ suggestion, together with media influences and broader socio-cultural expectations are proposed as central in the creation of DID (Lynn et al., 2012; Boysen and VanBergen, 2013). Such role-enactment is not considered as deception or faking, but as arising spontaneously with little or no conscious effort (Spanos, 1994; Lilienfeld et al., 1999; Boysen and VanBergen, 2013). However, given this focus on suggestion and enactment, a discourse around attention seeking behavior easily infiltrates this perspective and further polarizes the debate. Spanos (1994, p. 143), for example, states that “[i]n the last 20 years, the notion of multiple personality disorder has become commonplace in North American culture and is now a legitimate way for people to understand and express their failures and frustrations, as well as a covert tactic by which they can manipulate others and attain succor and other rewards.” McHugh (1993) considers the behavior of DID patients as a means to validate a sick role, which in turn provides opportunities for the patient to gain attention and satisfaction. Blaming the patient is not removed here and unsurprisingly these assertions have given rise to strong opposition.

Despite these provocative statements, SCM proponents make a strong case for the iatrogenic nature of DID. Their main arguments include the observation of important differences in the nature and prevalence of DID across time and cultures, the strong link between treatment and DID diagnoses with almost no diagnoses outside of treatment (in addition to an increase in the number of alters in the course of treatment), and the uneven distribution of DID cases across clinicians (Lilienfeld et al., 1999; Boysen and VanBergen, 2013; Lynn et al., 2014).

A close reading of the literature also shows that they generally do not discard the complexity of the underlying problems (Lynn et al., 2012, 2014) and acknowledge a pre-existing problem, as illustrated by Spanos (1994): “the SCM does not deny that much of the psychopathological raw material from which DID is sculpted exist prior to professional intervention.” However, this nuance appears to get lost in the heat of the discussion, where the idea that the therapist might be to blame gets entangled in the use of the term ‘iatrogenic.’

Following this model, the importance of traumatic experiences in the history of the patient could be rejected more often than necessary. Nevertheless, as Lilienfeld et al. (1999) argue, while the PTM and the SCM have presuppositions that are not necessarily mutually exclusive, they diverge quite substantially with respect to the main explanation for the emergence of alters. However, the SCM does not altogether discard the possibility of trauma as a predisposing factor (e.g., for increased proneness to fantasy and receptivity for the therapist’s suggestion), yet it does not accept the idea that the creation of alter identities is a direct defensive reaction to the experience of trauma. The PTM, on the other hand, does not altogether reject the possibility of iatrogenic factors playing a role for some DID patients; yet, no more than in other pathologies and not as a central mechanism. Nevertheless, the main difference at the level of trauma as a specific and direct causal factor remains a fundamental issue with far-reaching implications for treatment.

In the end, arguing about whether trauma is a direct or indirect etiological factor for specific symptoms only distracts us from an essential issue related to the underlying dynamics that might prove more useful in conceptualizing treatment for these disorders. This boils down to safeguarding a place for the subject, which is crucial for any understanding of diagnostics or treatment. Unfortunately, the subject as *subject*, rather than merely victim, seems to be precisely what disappears into the background in the trauma model. Interpreting complex pathology like DID strictly in terms of childhood (sexual) trauma provides an encompassing explanation (Libbrecht, 1995), yet distracts us from the particular meaning that symptoms have for the subject. Subjective meaning should, however, be at the center of diagnosis and treatment (Vanheule, 2014).

Below we will explore the potential of using a Freudian–Lacanian perspective on (1) the role of trauma in identity development; (2) the hysterical subject structure in order to understand dissociative symptoms and DID.

In the first part, it is proposed that examining the development of human identity with its fundamental dividedness, might help us to understand on a more structural level how dissociative phenomena can arise in the face of traumatic experiences. This relates to the question of etiology, which is the focus of the PTM. Given that there is no unique or specific link between certain traumatic experiences and dissociative symptoms (Bistoen et al., 2014), the PTM cannot explain adequately why traumatic events might lead to dissociation. We argue that the notions of structural and accidental trauma and their relationship to identity formation can help to understand the link between trauma and dissociation.

In the second part, we bring in the notion of structure as a necessary dimension to further understand this link and the specificity of DID as it appears in current treatment approaches.

In this context, I argue that the reigning discourse on DID could provide a mold into which the hysterical subject fits his/her suffering. Considering the perspective of hysterical dynamics might help to understand how iatrogenic influences can shape the specificity of the spectacular symptomatic tableau of DID. I argue that by focusing solely on the symptom (of alters) in the therapeutic process we may further alienate the subject and preclude him/her from coming to terms with the irremediable dividedness of human psychic functioning.

While the SCM seems to consider the possibility of a shared underlying psychopathological configuration running through the different appearances of dissociative states over time and across cultures (Spanos, 1994; Lilienfeld et al., 1999), it unfortunately does not explore such subjective dynamics any further. Instead, it focuses on invalidating the diagnosis itself and ultimately proving the PTM wrong. Consequently, and even despite all intentions (see Lilienfeld et al., 1999), any dialog with the PTM gets stuck on the question of whether or not DID is real. When we go back one century to the phenomenon of hysteria, where the idea of multiple personality arose, we see precisely the same questions surrounding (conversion) hysteria at that time. There were believers and disbelievers, those that considered hysteria a righteous disease and those that considered these patients as malingerers.

We will not get distracted by the question as to whether DID or hysteria are real here: Whereas the presence of traumatic experiences cannot serve as the linchpin of DID’s ontological status, accepting the psychogenic or iatrogenic nature of a condition cannot refute it. In brief, even if suggestibility is central to understanding hysterical symptoms, it does not mean that they are not real symptoms (see also Hacking, 1992). Suggestibility, or what the SCM call fantasy proneness, implies that therapists and more broadly the socio-cultural context (might) influence the way symptoms appear and evolve, and this influence might be detrimental rather than beneficial. While the SCM follows this logic in its attempt to understand possible influences that may lie at the origin of DID, it does not elaborate this further. However, considering this in relation to a fundamental underlying structure might provide more fruitful ways to consider diagnostic and treatment issues.

While the link between multiple personalities and hysteria was lost in the latter half of the twentieth century, when the DSM’s descriptive and symptomatic approach to diagnosis came to dominate, we should not forget that it was within theories on hysterical mechanisms that the idea of dissociation came to the fore: in short, dissociation was considered as the basic mechanism in hysteria (Janet, 1889/1973, 1949/2006; Breuer and Freud, 1895/1955). Before exploring this further, I first elaborate the idea of the divided subject as a possible framework to understand dissociative symptoms on a more structural level.

## Exploring the Link Between Trauma and Dissociation: A Freudian–Lacanian Approach to the Becoming of the Subject

Already in its terminology, the diagnosis of DID situates the problem that these patients experience at the level of identity, or, in other words, in the experience of the self, of being me. Every human being probably recognizes the experience of reacting, behaving, or feeling in a way that conflicts with our idea of who we are, our identity. In DID, however, these reactions, behaviors, and feelings appear to be split off to such an extent that they are experienced as belonging to another ‘me’ or not even experienced consciously at all.

From a psychoanalytic perspective, identity is constructed in an interactive process with important others and is strongly related to drive regulation (Vanheule and Verhaeghe, 2009). Freud conceptualized this process as the structural trauma, the nucleus of repression as the drive can never be entirely represented psychically. For Freud, the drive was to a certain extent inherently traumatic.

Freud situates the development of the Ego in identificatory processes following object-cathexes (Freud, 1923/1975). For Freud, the inauguration of the Ego firstly involves primary identification with primary caregivers^2^ and on the basis of this, subsequent, secondary layers of identification. This implies that the Ego is divided between these layers despite being experienced as a coherent unity.

In neurosis, this division at the base of the ego is the source of conflict and of repression, comprising the inauguration of the unconscious and the fundamental division in human psychic functioning. In this context, Freud (1923/1975) even explicitly refers to the phenomenon of multiple personality when saying that

“Although it is a digression from our aim, we cannot avoid giving our attention for a moment longer to the ego’s object-identifications. If they obtain the upper hand and become too numerous, unduly powerful and incompatible with one another, a pathological outcome will not be far off. It may come to a disruption of the ego in consequence of the different identifications becoming cut off from one another by resistances; perhaps the secret of cases of what is described as ‘multiple personality’ is that the different identifications seize hold of consciousness in turn. Even when things do not go so far as this, there remains the question of conflicts between various identifications into which the ego comes apart, conflicts which cannot after all be described as entirely pathological (pp. 30–31).”

Lacan elaborated this theory in his ‘return to Freud’ and put the relation with the other at the center of identity formation and the becoming of the subject. Early in his teaching, his work on the mirror stage was used to show that the basis of our identity concerns mirroring processes and identifications with what is offered by the other (Lacan, 1949/2006, 1953-4/1988). He situates the identification with the body image as the first layer of the Ego: The infant identifies with the (mirrored) image and in that way gains mastery over its chaotic bodily experience and the disturbance initiated by the drives. Whereas animal development could be considered as entirely regulated by the image (the Imaginary order), the human subject must be integrated into the symbolic order, mediated by the words of others, the signifiers introduced (i.e., the words, behaviors and gestures of the other that progressively informs the infant of its bodily coherence and unity; the Symbolic order). This process, with which the child increasingly invests, introduces the child into what Lacan calls the whole ‘treasure trove of signifiers’ available in the broader (immediate and cultural) linguistic context in which it must live (Lacan, 1958/2006). Lacan (1954-5/1988) formalized this with his concept of the big Other, which denotes both specific others and language as the basis of our psychic worlds. As identity is based on the primary and secondary identifications with the images and words coming from the outside, our sense of self must be considered as fundamentally alienated, coming from or incorporated from the Other (Lacan, 1955/2006). From this theoretical framework, we can see how human identity is fundamentally unstable. The split or inevitable conflict between different identificatory layers implies that the subject is essentially divided: there is no ultimate or true self to be found, only a division at the core of our being (Lacan, 1960/2006).

For Lacan, when the subject enters the realm of language something is gained, a more or less coherent sense of self and mastery over the chaotic nature of the drive and the enigmatic relation to others. At the same time something is lost, a leftover that remains which cannot be translated into language.

Here, we can bring in Lacan’s concept of the Real (Lacan, 1954-5/1988, 1957/2006, 1964/1998): For Lacan the Real, is by definition traumatic for the subject as it comprises that which cannot be represented psychically (Freud’s structural trauma). This is reminiscent of Freud’s definition of what makes up a traumatic experience, namely an experience of excess tension that overwhelms the Ego such that it fails to process the excitation psychically (Freud, 1917/1978), where we see a certain parallel between the traumatic nature of the drive and traumatic experiences. Whereas the latter is an external danger and is accidental (not necessarily so), the former is an internal threat that is structural (necessarily so for each human subject)^3^.

Following Freud, Lacan situates an unbridgeable gap at the core of our being. Identity formation (based on successive identifications with images and words) consists of life-long process of managing that gap, covering it, as language always falls short of fully representing the drive.

Thus, if the primary social bond with caregivers is characterized by accidental trauma (i.e., external trauma), the internal (structural trauma) will be interwoven with it and will have detrimental effects on the symbolic-imaginary structure that makes up our identity. In other words, if the other that we depend on to gain control over our internal experience, if the mirroring is itself traumatic and inconsistent, the resulting process of drive regulation and identity formation will remain chaotic.

We could say that in a more or less stable system, conflict between identificatory layers leads to repression and symptom formation, in a less stable system it set the ground for a potentially more radical dissolution of the self-experience. Here, we might situate the relationship between trauma and dissociation vis à vis the relationship with the Other. It seems that in DID patients, where the trauma is mostly situated in childhood interpersonal relationships (Schimmenti and Caretti, 2016), the resulting loose self-experience or precarious symbolic-imaginary envelope of the Real gives rise to a more raw and unprocessed appearance of the division of the subject.

However, as this underlying division is structural and not curable, this dividedness at the core of our being is at the basis of all symptom development. In that sense, it is not surprising that a number of authors consider dissociation as present in every subject and conceptualize a continuum of dissociation (Price, 1987; Butler, 2006).

Moreover, as it is the subject who must come to terms with his condition, it is thus counterproductive to approach the patient as victim, since this precludes the possibility of taking responsibility, which is the necessary precondition for all change (Herman, 1992).

## The Versatile Nature of DID Symptoms: The Hysterical Subject Position

Above I elaborated how we might understand the link between trauma and dissociation within the development of the subject in relation to others. This broad view on identity formation sheds light on the complexity of trauma and how it might affect human psychic functioning. However, numerous arguments proposed by the SCM in questioning the veracity of the DID diagnosis concern the changeable nature of DID symptomatology over time. As indicated above, it is arguable that the specificity of the DID pathology (the way these symptoms appear and change over time and context) might be understood when situated within a hysterical subject structure^4^.

Our exploration of the fundamental dividedness of the subject in no way means to question the seriousness of the pathology or even the role of trauma. However, it is clear that traumatic experiences appear to give rise to different types of (psychological) problems and we think the nature of these problems depends on the structural position of the subject on which they intervene. Moreover, specific societal and therapeutic discourses can further shape the form symptoms take.

Below I discuss two related characteristics of hysterical neurosis, again from a Freudian–Lacanian perspective, in order to reframe arguments put forward in the SCM: the subject’s relation to the Other and to knowledge.

From a psychoanalytic point of view, hysteria, as a clinical structure, refers to a specific subject position, and not to a specific set of symptoms (Fink, 1997). Following Freud, Lacan’s structural approach to human subjectivity and psychopathology allows us to look beyond specific symptomatology, as such, as symptoms can arise from very different psychical dynamics (Fink, 1997; Verhaeghe, 2004). From a Lacanian point of view, someone can be diagnosed as hysteric without any of the so-called typical hysterical symptoms: Indeed it is precisely the inconsistency of hysterical symptomatology that characterizes this clinical structure, and this is by virtue of its specific positioning toward the Other.

As discussed above, the subject’s position, its identity, takes form in relation to others, firstly in relation to the primary caregiver through mirroring and the process of identification. When the child turns to his caretakers, it expresses a demand that goes beyond the drive-related need to express at the same time a demand for love, recognition (Rogers, 2007). In turn, the caregiver signifies the needs of the child, thereby placing these needs within a certain framework; they demand that the child behaves in certain ways, inscribes itself into the linguistic order that is offered. The child attempts to read what it is that the parent wants from them in order to gain love and affection. In that sense, the primary caregiver’s desire becomes the source of our own desire. Such early interpretations about what the Other wants will more or less generate a blueprint, which comes into play within each subsequent relationship, and thus also in the therapeutic relationship with the therapist as other.

This process and the way the Other is perceived is a complex bidirectional interactional process where subject and other are hardly distinguishable, and can be characterized for the hysterical subject position by a focus on fusion (in contrast with separation for the obsessional neurotic subject). He or she attempts to meet the desire of the Other, yet interprets the message of the Other in the sense that what is given is not enough, that the subject does not meet the desires of the Other. It is the Other who is the desiring subject and it is the hysterical subject who appropriates to this desire by identifying with it. As the desire of the Other is also grounded in a structural lack, the hysterical subject gets trapped in a never ending attempt to complete the Other after all. This dominant tendency to identify with and adapt to the other’s desire, which is part and parcel of the subject’s development itself, allows us to understand the myriad ways symptoms can appear (Verhaeghe, 2004). Therefore it is crucial to consider this basic relationship to the Other as a focus of both diagnostic and therapeutic approaches, rather than starting from the appearance of the symptom.

In terms of James, we see his never ending attempt to comply with the demands of, for example, his girlfriend in order to be loved, to be the man she desires, thereby sacrificing his own subjectivity. This implies that what does not fit the picture in his experience of his girlfriend’s desire is repressed. Repression is a central mechanism in hysterical symptom formation, repression of conflicting wishes and desires of those thoughts in which one cannot recognize oneself. Moreover, the hysterical symptom is pre-eminently a compromise formation between such conflicting desires (Freud, 1900/1978), which in the end go back to different layers of identification with opposing desires of others. This illustrates par excellence that the human subject, human psychic functioning is not one consistent whole, but is essentially divided (Lacan, 1957-8/1998; Fink, 1996). The subject is divided between different desires of important others, between his own conscious and unconscious and so on.

Following this logic, in dissociation we can see these conflicting tendencies literally appear in what seems like two apparently different persons, yet it is the division that cannot be experienced by the subject that causes the conflicting desires to be split off. This splitting of psychic activity was considered by Breuer and Freud (1895/1955) in their famous *Studies on Hysteria* as the basic mechanism of hysterical neurosis. In the case of James, we see how his sexual urges that are not in line with his girlfriend’s desire, his own anger that he cannot allow himself to experience, show up in his dissociative states^5^.

In his/her unstoppable quest to appropriate the desire of important others, the hysterical subject turns to others that are imbued with knowledge, another that is expected to know. This dynamic between subject and Other is what Lacan formalizes in his discourse theory with the hysterical discourse (Lacan, 1964/1998). The subject, as divided, addresses the Other as master in search of a signification of her lack, of what (s)he is missing. The specific others that are addressed are others in a position where knowledge is expected to reside. These positions will change over time. While priests incarnated the position of knowledge until the end of the 19th century, they were replaced or complemented by medical doctors and in a later phase psychotherapists. The hysteric turns to these others and identifies with the answer that is provided, the signification gained for their suffering. Because the lack, the underlying division, is structural and language can never fully grasp the Real of the drive, every answer produced by another will by definition always be incomplete. In that sense, identity not only consists of a never-ending process of successive identifications, but the process takes place in relation to a series of different master figures replacing each other.

Although the knowledge produced by another (such as a diagnostic label) never covers the truth underlying the subject’s dividedness, this does not preclude that the subject identifies with the answer, with the knowledge produced by the other placed in the position of the master.

## A Reappraisal of the Arguments

This concise and necessarily reductionist outline of a structural view on hysteria delineated the susceptibility of the hysterical subject for the desire of the Other, on the one hand, and the fundamental division of neurotic psychic functioning^6^, on the other hand. This allows us to understand and reinterpret the findings put forward by the SCM proponents as arguments for an iatrogenic nature of DID symptoms and leads the debate away from the question of the reality of these spectacular symptoms.

An important argument concerns the major fluctuations over time and across cultures in the reporting of DID cases (Spanos, 1994; Piper and Merskey, 2004). Whereas there were a number of cases reported around the turn of the 19th to the 20th century, there were hardly any case reports between 1920 and 1970. However, since then the number of reported cases has increased exponentially (Merskey, 1995; e.g., Kluft, 1988; Putnam, 1989; Ross, 1997) and the large increase in reported cases closely followed the publication of the bestseller ‘Sybil,’ a young woman with a history of childhood abuse who manifested 16 alter identities (Lynn et al., 2012). Attention for DID in the literature thus comes in waves, rather than receiving a steady amount of research and theoretical attention. More recently, Pope et al. (2006) and Boysen and VanBergen (2013) tracked publication rates from 1984 to 2003 and from 2000 to 2010, respectively. They note a sharp peak in publications on DID in the late 1990s followed by a steep decline since that time (Pope et al., 2006). In the first decennium of the 21st century, research productivity was slow but steady, focusing mainly on descriptive studies concerning the clinical features of the disorder (Boysen and VanBergen, 2013) and its treatment (e.g., Brand et al., 2009). From the point of view of the hysterical subject structure, it is not so difficult to understand that literature and media attention influences prevalence numbers. This does not imply that there should be an increase in psychopathology in general. However, the color of the hysterical symptoms, the terms used to signify mental suffering and the experience of conflict is guided by the reigning discourse.

The appearance of alters also changed over time. Whereas it was very rare that more than two or eventually three personality states were described in the early 20th century (Merskey, 1995; Baeten, 1998), more recently an average of 13 alters is reported (Putnam et al., 1986; Putnam, 1989) with extremes of up to 4000 alters (Kluft, 1988, 2006). Moreover, today alters seem to shift immediately and without warning, whereas in the 19th and early 20th century a transitional period (e.g., of sleep; Hacking, 1991; Spanos, 1994) was mostly described. Also the secondary states were regularly not described as equaling the common conscious state of mind, yet rather appear as somnambular or hypnotic states. This is clear in descriptions of Azam (1887, in Merskey, 1995), who explained dual personality in terms of a doubling of consciousness connected to somnambulism. In Breuer’s and Freud’s famous case of Anna O. (Breuer and Freud, 1895/1955), the secondary personality state is described as linked to auto-hypnosis, a state of altered consciousness. In sum it seems that dual personality states were linked to dual consciousness, termed ‘double conscience’ by Janet (1889/1973), which he considered to be a fundamental hysterical mechanism.

When considering the chameleon like nature of the hysterical subject (Verhaeghe, 2004), these observations are not so hard to understand. As societies and cultural discourses change, so too do symptoms. The hysterical subject adapts to the discourses on mental and physical illness in the medical world and the broader society. The hysteric fits his/her suffering into the mold provided by the reigning master discourse. Again, this in no way means we consider this to be a conscious process. Rather, these identificatory processes are dominant in the psychic make-up and proceed on an unconscious level, only surfacing in instances where the subject feels fake, not knowing who she is herself and sensing the major impact of the Other on her identity (Fink, 1997).

Not only does the hysterical subject identify with the reigning discourse, she actively turns toward the presumed master to signify her suffering. Doctors and therapists on account of their professional status easily occupy the position of the master who knows. In this respect, therapists and researchers stepping on the barricades as advocates of the veracity of the DID diagnosis and its traumatic origin preeminently incarnate such a position.

What is remarkable, and in line with our argument, is that most DID cases are reported in North America and center around a limited number of therapists and research groups. While Spanos (1994) for example notes that the majority of cases are reported by a small number of therapists, Gleaves (1996) argues that these research teams used data from a large number of clinicians. As Lilienfeld et al. (1999) indicates, however, there is a clear selection bias with questionnaires sent out to clinicians that are members of associations concerning dissociation, or expressing a clear interest in the phenomenon. So it seems, therapists group around a fascination for these symptoms, form associations and communicate around this pathology. It is not so strange starting from the reasoning we are laying out here to understand why these therapists or groups of therapists find such high prevalence numbers, numbers that are not found anywhere else. The hysteric subject tends to search for someone that can incarnate the role of a master figure to find an answer to what (s)he is missing and models symptoms in line with these master’s expectations (again, not in a conscious way, but as the basic mechanism of psychic functioning). The therapist/master consequently finds what he expects and sees his theory confirmed. It is clear from the literature that a suggestive dimension is strongly present in the work of therapists ‘believing’ in the idea of DID and its direct relationship with trauma (e.g., techniques for mapping alter systems, setting up communication between alters by means of notes, blackboards, agreements). This dimension of suggestion is further supported by the observation that in most cases alters only appear within the context of therapy and almost never as an admission complaint. PTM proponents argue that DID is mostly hidden and that patients are unwilling to show their symptoms because of shame, for example. They claim that it requires a skillful therapist to notice the signs of DID. Descriptions of both diagnostic and therapeutic procedures show that they actively probe for alters, for example by asking if someone is else speaking when a patient reacts or talks a bit differently than usual (e.g., more assertive) (Kluft, 2006). Starting from their fundamental psychic make-up, the hysterical subject hears this as a suggestion (and rightly so) to which they consent by identifying with the suggested symptom. Surely, this gives form to an existing problematic founded on the essential dividedness from which they are suffering. In that sense, the suggestive nature of questions actively addressing different personalities or alters within a patient does not create something *ex nihilo*. Rather, it substantiates the fundamental dividedness of the subject’s identity that is precisely scattered across different identificatory layers that are often in contradiction to one another and cause intra-psychic conflict. But we might wonder whether this division is not intensified and pathologized further by substantiating alter identities this way. We could say that it is strange that such spectacular symptoms like DID go unnoticed by relatives and other clinicians across the world, clinicians with years of experience working with complex pathology and traumatic experiences. On the other hand, as these symptoms find their basis in conflicting desires and identifications, dissociative tendencies are not unexpected in the hysteric and serve the function of appropriating the desire of the Other. Again, the question is whether therapists should further consolidate these tendencies.

Therapeutic guidelines for working with DID patients mainly start from a trauma model and put a lot of emphasis on working with alter identities (International Society for the Study of Trauma and Dissociation [ISSTD], 2011). What appears to happen in therapy from a PTM point of view is that one ‘personality’ is considered to be truer than the others. Although treatment guidelines state that it is counter-therapeutic “to treat any alternate identity as more real or more important than any other” (International Society for the Study of Trauma and Dissociation [ISSTD], 2011, p. 132), their discourse clearly privileges certain alters and is dotted with terminology reifying alter personalities. For example, it is this ‘better’ person with whom literally agreements are made to control bad or dangerous alters (‘Safety agreements,’ International Society for the Study of Trauma and Dissociation [ISSTD], 2011). Also the focus on identifying alters and actively “helping them to be aware of one another as legitimate parts of the self… (p. 132)” substantiates the idea of alters and fosters the splitting off of these unbearable experiences. Such a practice essentially misrecognizes the fundamental and irremediable division of the subject that is so central in Lacan’s theory. Actually, such a practice boils down to what Lacan (e.g., Lacan, 1954-5/1988, 1958/2006) criticized in ego-analytic approaches where the analyst is supposed to ally with the good part of the ego to concur the bad parts. For Lacan (1958/2006) this leads to an adaptive practice where the analyst/therapist is the measure of all things.

This brings us to another way therapists might facilitate the creation of dissociative symptoms besides the role of suggestion and identification with the therapist’s desire, namely the intensification of repression and related dissociative tendencies by misrecognizing important parts of subjectivity. As shown in Lasky’s (1978) case of a woman presenting alter identities after at least 50 h of therapy, the misrecognition by the therapist of aggressive fantasies and a disturbed relational life pushes the patient to acting it out. Mrs. G. is a black American woman in her mid-thirties who consults Lasky for continual anxiety and recurrent bouts of depression. He describes her and states that he probably wanted to keep seeing her as “a charming and engaging woman who in spite of what seemed to be a neurotic depression was able to function in many ways which were admirable” (p. 368). In the first phase of the therapy, Lasky describes the focus of the therapy as rather concrete, discussing domestic problems and her history as one of ten children in a religious family. By around the 50th session, the strong ambivalence toward her mother because of an extramarital affair gradually came to the fore. Re-experiencing feelings of rage started to give rise to another way of appearing in the sessions. When the therapist noted this, she started to put these utterances in the mouth of someone else, who she named ‘Candy.’ After a very intensive session, she appears the next session to be behaving in a radically different way, dressed like someone else. When Lasky, surprised by this change, told her he had not seen this side of her before, she responded, ‘Get the shit out of your ears’ (p. 369). Lasky does not question the splitting tendencies in her psychic make-up, yet he does reflect on his own “wish to see her as well-integrated and not seriously disturbed” (p. 368) and relates this to the appearance of ‘Candy’ as a different personality. Where Lasky, as therapist, is able to reflect on his own desire to see her as a strong woman who functions well, given the difficult circumstances, he allows her to find words for the aggressive desires and the splitting disappears.

In sum, I think that in order to achieve memory integration it is crucial to create space for the full spectrum of subjectivity rather than focusing on alter identities directly. Indeed, we believe that the latter approach simply strengthens the dissociative process and prompts new alters to appear during the therapeutic process (e.g., Kluft, 2006). Oddly enough, it seems that in the literature on treatment for DID (e.g., Kluft, 2006; International Society for the Study of Trauma and Dissociation [ISSTD], 2011), addressing alters is considered the only way to give space for the entire field of subjectivity. However, as we argue here, it seems to create the opposite: whole parts of subjective experience are rendered hidden and repressed, as focusing on alters supports the notion that they are bad and should not be part of the subject’s experience.

## Conclusion

Considering both the PTM and the SCM in detail, I actually argued that both models hint at important mechanisms to understand DID: the role of trauma on the one hand and the role of the underlying subjective structure on the other. However, while both models are busy arguing against each other, they miss the opportunity to elaborate this fully. From a psychoanalytic point of view we could say they are talking about two sides of the same coin, i.e., two sides of subject formation, namely trauma and structure; yet without realizing how this might provide the possibility to bridge the gap between the two models.

Starting from the discussion above, I agree with the SCM’s view that prevailing discourses on DID and its related diagnostic and therapeutic approaches facilitates the phenomenon of DID and leads to an imaginary proliferation of alters, as illustrated by observations of DID patients with over 4000 alters (Kluft, 1988).

However, in line with the argument on structural trauma at the core of identity formation, we do not deny the real basis for this pathology, as the symptoms seem to present an enlargement of the structural division of human psychic functioning. Different identificatory layers of human identity often depict contradictory desires where conflicts inevitably arise. Such conflicts are difficult to experience by the subject and fall prey to repression. Traumatic contexts can induce painful identifications: in such situations the subject tries to understand the desire of the Other in an attempt to meet expectations and thus identifies with desires that are often more disruptive when the other is abusive, aggressive, transgressive… Conflicting desires exist for every neurotic subject, but the extent to which they derail the subject will always differ.

To avoid these disruptive desires and thus fail to appreciate the fundamental division between opposing desires will intensify and fixate conflict rather than help the subject come to terms with them. As illustrated by Lasky’s case study, we argue that it is crucial for the therapist to understand these so-called alters as reflective of different unconscious desires and thus as belonging to the subject, rather than considering them to be unreal parts of the self that must be overcome in lieu of the good and more real parts. This will create a space for the subject to give words to these experiences and assume them, rather than push the subject to act these tendencies out.

Rather than focusing on and being fascinated by the symptom itself, we must allow the subject to speak freely and not recoil from the darker side of his unconscious desire. This will help the patient put it into words and will have a therapeutic effect on symptoms, such that they dissipate. As history shows, to focus solely on the symptom of DID leads to the invention of all kinds of techniques (e.g., mapping of alter systems), which we argue fosters the symptom at the expense of the subject’s desire. Perhaps we should keep in mind Lacan’s (1953/2006) seemingly simple adage that “Psychoanalysis has but one medium: the patient’s speech” (p. 206) and trust on the power of this speech that is truly heard by an-Other to reveal the subject’s truth.

## Ethics Statement

This study was approved by the Ethical committee of Ghent University Hospital (Registration number: B670201318127). A clinical vignette from a larger study is briefly discussed. All patients receive oral and written information on the study and provide written informed consent.

## Author Contributions

The author is responsible for the entire manuscript. Other contributions are mentioned in the acknowledgements.

## Conflict of Interest Statement

The author declares that the research was conducted in the absence of any commercial or financial relationships that could be construed as a potential conflict of interest.

## Footnotes

1 This does not take away that in research as well as in clinical practice it is common for people who lived in abusive childhood situations to describe dissociative experiences (e.g., leaving their bodies and watching from the ceiling) during the abuse. This also allowed them to maintain a relationship with the abuser – who is often a primary caregiver – outside the horror of the abuse itself. In that sense, dissociation is understood as a defensive mechanism serving a protective function (Herman, 1992; Schimmenti and Caretti, 2016).

2 Freud (1923/1975) situates this in close relation to the dissolution of the Oedipus complex and these primary identifications form the basis of the Super Ego.

3 In that sense, the often heard critique that Freud dismissed the traumatic origin of hysteria and replaced it with fantasmatic structures is short-sighted. Indeed, at a certain moment in his work he questioned the reality of the sexually traumatic experiences, not to disregard the existence of traumatic experiences, but to indicate that the question of reality was not primary given the inherently traumatic dimension of human sexuality. In both cases the impossibility to represent an experience (whether internal or external) that requires mental processing is central.

4 It is clear that the notion of hysterical subject structure in the work of Freud, Lacan, and other psychoanalytic authors should be conceived as a broad notion designation a subject’s position within the neurotic spectrum and in itself does not refer to pathological states. In this context, however, and in the light of what was discussed above, we more specifically refer to subjects that qua personality structure can be considered hysteric, but where the structure given the (traumatic) developmental context does not provide stability.

5 It should be mentioned here, that dissociative symptoms are not to be expected for each subject showing a hysterical subject structure. It is only given certain contextual factors (cf. the interaction between structural and accidental trauma) that we can understand the lack of stability in the psychic system and the insufficient working of ‘ordinary’ repression.

6 i.e., the ‘normal’ human disposition. Neurotic in this sense does not imply any statement about pathology or the absence thereof.

## References

[B1] American Psychiatric Association (1968). *Diagnostic and Statistical Manual of Mental Disorders*, 2nd Edn Washington, DC: American Psychiatric Association.

[B2] American Psychiatric Association (1980). *Diagnostic and Statistical Manual of Mental Disorders*, 3rd Edn. Washington, DC: American Psychiatric Association.

[B3] American Psychiatric Association (2000). *Diagnostic and Statistical Manual of Mental Disorders*, 4th Edn-TR Washington, DC: American Psychiatric Association.

[B4] American Psychiatric Association (2013). *Diagnostic and Statistical Manual of Mental Disorders*, 5th Edn Washington, DC: American Psychiatric Association.

[B5] Baeten S. (1998). *Van Bezetenheid tot Dissociatie.* Leuven: Garant.

[B6] Bistoen G. Vanheule S. Craps S. (2014). Nachtraglichkeit: a Freudian perspective on delayed traumatic reactions. *Theor. Psychol.* 24 668–687. 10.1177/0959354314530812

[B7] Boysen G. A. (2011). The scientific status of childhood dissociative identity disorder: a review of published research. *Psychother. Psychosom.* 80 329–334. 10.1159/000323403 21829044

[B8] Boysen G. A. and VanBergen A. (2013). A review of published research on adult dissociative identity disorder: 2000-2010. *J. Nerv. Ment. Dis.* 201 5–11. 10.1097/NMD.0b013e31827aaf81 23274288

[B9] Brand B. L. Classen C. C. McNary S. W. Zaveri P. (2009). A review of dissociative identity disorders treatment studies. *J. Nerv. Ment. Dis.* 197 646–654. 10.1097/NMD.0b013e3181b3afaa 19752643

[B10] Breuer J. Freud S. (1895/1955). *Studies on Hysteria.* The Standard Edition Vol. 2 London: Hogarth Press, 1–335.

[B11] Butler L. D. (2006). Normative dissociation. *Psychiatric Clin. North Am.* 29 45–62. 10.1016/j.psc.2005.10.004 16530586

[B12] Dalenberg C. J. Brand B. L. Gleaves D. H. Dorahy M. J. Loewenstein R. J. Cardena E. (2012). Evaluation of the evidence for the trauma and fantasy models of dissociation. *Psychol. Bull.* 138 550–588. 10.1037/a0027447 22409505

[B13] Fink B. (1996). *The Lacanian Subject.* Princeton, NJ: Princeton University Press.

[B14] Fink B. (1997). *A Clinical Introduction to Lacanian Psychoanalysis. Theory and Technique.* Cambridge: Harvard University Press.

[B15] Freud S. (1900/1978). “The interpretation of dreams,” in *The Standard Edition of the Complete Psychological Works of Sigmund Freud*, Vol. 4–5 (London: Hogarth Press), 1–626.

[B16] Freud S. (1917/1978). “Introductory lectures on psycho-analysis PART III,” in *The Standard Edition of the Complete Psychological Works of Sigmund Freud*, Vol. XVI (London: Hogarth Press), 273–285.

[B17] Freud S. (1923/1975). “The Ego and the Id,” in *The Standard Edition of the Complete Psychological Works of Sigmund Freud*, ed. Strachey trans. J. Vol. 19 (London: Hogarth Press), 1–66.

[B18] Gleaves D. H. (1996). The sociocognitive model of dissociative identity disorder: a reexamination of the evidence. *Psychol. Bull.* 120 42–59. 10.1037/0033-2909.120.1.42 8711016

[B19] Hacking I. (1991). Double consciousness in Britain 1815-1875. *Dissociation* 4 134–146.

[B20] Hacking I. (1992). “Multiple personality disorder and its hosts,” in *History of the Human Sciences*, Vol. 5. London: Sage, 3–31.

[B21] Herman J. (1992). *Trauma and Recovery: The Aftermath of Violence - from Domestic Abuse to Political Terror.* New York, NY: Basic Books.

[B22] International Society for the Study of Trauma and Dissociation [ISSTD] (2011). Guidelines for treating dissociative identity disorder in adults, third revision. *J. Trauma Dissociation* 12 115–187. 10.1080/15299732.2011.537247 21391103

[B23] Janet P. (1889/1973). *L’automatisme Psychologique: Essai de Psychologie Experimentalesur les Formes Inférieures de l’activité humaine.* Paris: Société Pierre Janet.

[B24] Janet P. (1911/1983). *L’état Mental des Hystériques.* Marseille: Lafitte reprints.

[B25] Kluft R. P. (1988). The phenomenology and treatment of extremely complex multiple personality disorder. *Dissociation* 1 47–58.

[B26] Kluft R. P. (2006). Dealing with alters: a pragmatic clinical perspective. *Psychiatr. Clin. N. Am.* 29 281–304. 10.1016/j.psc.2005.10.010 16530598

[B27] Lacan J. (1949/2006). “The mirror stage as formative of the function of the I as revealed in psychoanalytic experience,” in *Écrits: The First Complete Edition in English*, Fink trans. B. Grigg R. (New York, NY: W. W. Norton & Company), 75–81.

[B28] Lacan J. (1953-4/1988). *The Seminar of Jacques Lacan, Book I: Freud’s Papers on Technique.* London: W.W. Norton.

[B29] Lacan J. (1954-5/1988). *The Seminar of Jacques Lacan, Book II: The Ego in Freud’s Theory and in the Technique of Psychoanalysis.* Cambridge: Cambridge University Press.

[B30] Lacan J. (1955/2006). “The Freudian thing,” in *Écrits: The First Complete Edition in English*, Fink trans. B. Grigg R. (New York, NY: W. W. Norton and Company), 334–363.

[B31] Lacan J. (1957-8/1998). *Le Séminaire, Livre V: Les Formations de l’inconscient [The Seminar, book V: the formations of the unconscious].* Paris: Seuil.

[B32] Lacan J. (1957/2006). “Response to Jean Hyppolite’s Commentary on Freud’s Verneinung,” in *Écrits: The First Complete Edition in English*, Fink trans. B. Grigg R. (New York, NY: W. W. Norton & Company), 318–333.

[B33] Lacan J. (1958/2006). “The direction of the treatment and the principles of its power,” in *Écrits: The first Complete Edition in English*, Fink trans. B. Grigg R. (New York, NY: W. W. Norton & Company), 489–542.

[B34] Lacan J. (1960/2006). “The subversion of the subject and the dialectic of desire in the Freudian unconscious,” in *Écrits: The First Complete Edition in English*, Fink trans. B. Grigg R. (New York, NY: W. W. Norton & Company), 671–702.

[B35] Lacan J. (1964/1998). *The Seminar XI, The Four Fundamental Concepts of Psychoanalysis*, ed. Miller J.-A. Alan Sheridan trans. (London: W.W. Norton).

[B36] Lacan J. ed. (1953/2006). “The function and field of speech and language in psychoanalysis.” in *Écrits: The First Complete Edition in English*, Fink trans. B. Grigg R. (New York, NY: W. W. Norton & Company), 237–268.

[B37] Lasky R. (1978). The psychoanalytic treatment of a case of multiple personality. *Psychoanal. Rev.* 65 355–380. 104317

[B38] Libbrecht K. (1995). De kliniek van de meervoudige persoonlijkheid. *Psychoanal. Perspectieven* 26 45–60.

[B39] Lilienfeld S. O. Kirsch I. Sarbin T. R. Lynn S. J. Chaves J. F. Ganaway G. K. (1999). Dissociative identity disorder and the sociocognitive model: recalling the lessons of the past. *Psychol. Bull.* 125 507–523. 10.1037/0033-2909.125.5.507 10489540

[B40] Lynn S. J. Lilienfeld S. O. Merckelbach H. Giesbrecht T. van der Kloet D. (2012). Disocciation and dissociative disorders: challenging conventional wisdom. *Curr. Dir. Psychol. Sci.* 21 48–53. 10.1177/0963721411429457

[B41] Lynn S. J. Merkelbach H. Giesbrecht T. Loftus E. F. Garry M. Lilienfeld S. O. (2014). The trauma model of dissociation: inconvenient truths and stubborn fictions. comment on Dalenberg(2012). *Psychol. Bull.* 140 896–910. 10.1037/a0035570 24773505

[B42] McHugh P. R. (1993). Multiple personality disorder. *Harv. Ment. Health Newletter* 10 4–6.

[B43] Merskey H. (1995). *The Analysis of Hysteria. Understanding Conversion and Dissociation.* Glasgow: Bell & Bain.

[B44] Mulhern S. (1994). Satanism, ritual abuse, and multiple personality disorder: a sociohistorical perspective. *Int. J. Clin. Exp. Hypn.* 42 265–288. 7960286 10.1080/00207149408409359

[B45] Piper A. Merskey H. (2004). The persistence of a folly: a critical examination of dissociative identity disorder. Part I. The excesses of an improbable concept. *Can. J. Psychiatry* 49 592–600. 15503730 10.1177/070674370404900904

[B46] Pope H. G. Barry S. Bodkin A. Hudson J. I. (2006). Tracking scientific interest in the dissociative disorders: a study of scientific publication output 1984-2003. *Psychother. Psychosom.* 75 19–24. 10.1159/000089223 16361871

[B47] Price R. (1987). Dissociative disorders of the self: a continuum extending into multiple personality. *Psychotherapy* 3 387–391.

[B48] Putnam F. W. (1989). Diagnosis and treatment of multiple personality disorder. New York, NY: Guilford Press.

[B49] Putnam F. W. Guroff J. J. Silberman E. K. Barban L. Post R. M. (1986). The clinical phenomenology of multiple personality disorder: review of 100 recent cases. *J. Clin. Psychiatry* 47 285–293. 3711025

[B50] Rogers A. (2007). *The Unsayable: The Hidden Language Of Trauma.* New York, NY: Random House.

[B51] Ross C. A. (1997). *Dissociative Identity Disorder: Diagnosis, Clinical Features, and Treatment of Multiple Personality.* New York, NY: Wiley.

[B52] Sarbin T. R. (1995). On the belief that one body may be the host to two or more personalities. *Int. J. Clin. Exp. Hypn.* 43 163–183. 7737761 10.1080/00207149508409959

[B53] Sarbin T. R. (1997). Multiple personality disorder: fact or artifact? *Curr. Opin. Psychiatry* 10 136–140. 10.1097/00001504-199703000-00015

[B54] Schimmenti A. Caretti V. (2016). Linking the overwhelming with the unbearable: developmental trauma, dissociation, and the disconnected self. *Psychoanal. Psychol.* 33 106–128. 10.1037/a0038019

[B55] Spanos N. P. (1994). Multiple identity enactments and multiple personality disorder: a sociocognitive perspective. *Psychol. Bull.* 116 143–165. 10.1037/0033-2909.116.1.143 8078970

[B56] Spiegel D. Loewenstein R. J. Lewis-Fernández R. Sar V. Simeon D. Vermetten E. (2011). Dissociative disorders in DSM-5. *Depress. Anxiety* 28 824–852. 10.1002/da.20874 21910187

[B57] Vanheule S. (2014). *Diagnosis and the DSM: A Critical Review.* London: Palgrave Macmillan.

[B58] Vanheule S. Verhaeghe P. (2009). Identity through a psychoanalytic looking glass. *Theory Psychol.* 19 391–411. 10.1177/0959354309104160

[B59] Verhaeghe P. (2004). *On Being Normal and other Disorders: A Manual for Clinical Psychodiagnostics.* New York, NY: Other Press.

